# The Breaking Point: Family Dementia Caregivers’ Recognition of the Need to Intervene

**DOI:** 10.1093/geroni/igaa057.499

**Published:** 2020-12-16

**Authors:** Candace Harrington, Cheryl Dean-Witt, Frances Hardin-Fanning

**Affiliations:** 1 University of Louisville, Louisville, Kentucky, United States; 2 University of Louisville, Louisville,, Kentucky, United States; 3 University of Louisville, Winchester, Kentucky, United States

## Abstract

Family dementia caregivers are an under-recognized and valuable geriatric workforce whose services have broad implications for health care systems. Family dementia caregivers may experience uncertainty, loss of role identity, involuntary role assumption, or undesirable life transitions. Subsequent unintentional mistreatment or abuse of their family care recipient may occur. Approximately 50% of caregivers admit to some form of mistreatment of their loved one who lives with dementia. Using Selder’s (1989) life transition theory, this qualitative study explored family members’ life transition process toward their new role identities as family dementia caregivers to better understand the personal and historical contexts of caregiving. Semi-structured interviews were conducted with 10 participants to answer the questions: How does one acquire the role of primary family dementia caregiver?” and “How do personal and historical contexts inform the family dementia caregiver role?” Richness of data drove our sample size. Epistemological integrity ensured trustworthiness and rigor. A modification of Colaizzi’s (1978) analytic method was used for interpretative phenomenological analysis. The emergent themes uncovered by participants’ statements included: “It’s my turn”, “the breaking point”, and “a fine line” with the subtheme “balancing dignity and safety”. Participants described their introspective journeys toward a changed reality as family dementia caregivers. Our findings suggested the need for early recognition and vigilance to prevent the exploitation and mistreatment of those with dementia. Rural agriculture-based family caregivers in our study described unique and challenging characteristics. Further research is needed to explore the implications of these contextual nuances for rural agriculture-based family dementia caregivers.

